# The World Federation of Societies of Biological Psychiatry (WFSBP) consensus statement on biological markers for anorexia nervosa

**DOI:** 10.1192/j.eurpsy.2026.10379

**Published:** 2026-07-31

**Authors:** H. Himmerich, WFSBP Task Force on Eating Disorders

**Affiliations:** Institute of Psychiatry, Psychology and Neuroscience, King’s College London, London, United Kingdom

## Abstract

**Abstract:**

The World Federation of Societies of Biological Psychiatry (WFSBP) Task Force on Eating Disorders reviewed, summarised and evaluated the relevant literature on objectively measurable biological markers associated with anorexia nervosa (AN), and a consensus regarding the significance of the published evidence was reached.

Candidate biological markers that have been associated with AN include clinical, molecular, cellular, neuroimaging, digital, cardiac and neurophysiological parameters. Some clinical and laboratory parameters are risk markers in clinical practice. Biological markers have pathophysiological relevance in understanding the biological and metabolic pathophysiology of AN and its physical health consequences. Only few studies have examined pharmacogenetics or therapeutic drug monitoring as tools to monitor and guide the treatment of AN.

Studies on sensitivity, specificity and the prognostic value of these markers are lacking.

**Disclosure of Interest:**

None Declared

